# Progress in the study of parvovirus entry pathway

**DOI:** 10.1186/s12985-023-02016-z

**Published:** 2023-04-04

**Authors:** Jiuming Shi, Yifeng Pei, Qian Yu, Hao dong

**Affiliations:** 1 College of Life Sciences, Jilin Agricultural University, Changchun, 130118 Jilin Province China; 2 Engineering Research Center of Bioreactor and Pharmaceutical Development, Jilin Agricultural University, Changchun, 130118 China

**Keywords:** Parvovirus, Nuclear pathway, Imp β participation, Nuclear membrane permeability

## Abstract

A group of DNA viruses called parvoviruses that have significant effects on cancer therapy and genetic engineering applications. After passing through the cell membrane to reach the cytosol, it moves along the microtubule toward the nuclear membrane. The nuclear localization signal (NLS) is recognized by importin-beta (impβ) and other proteins from the complex outside the nuclear membrane and binds to enter the nucleus via the nuclear pore complex (NPC). There are two main pathways for viruses to enter the nucleus. The classical pathway is through the interaction of imp α and impβ with NLS via NPC. The other is the NPC mediated by the combination of impβ and it. While the capsid is introduced into the nucleus through classical nuclear transduction, there is also a transient nuclear membrane dissolution leading to passive transport into the nucleus, which has been proposed in recent years. This article mainly discusses several nuclear entry pathways and related proteins, providing a reference for subsequent research on viral entry pathways.

## Introduction

Parvovirus is a non-enveloped, single-stranded (ss) DNA virus. Clinically, it is mainly characterized by hemorrhagic and enteritis. Parvovirus circulates worldwide, such as in the 1978 global canine parvovirus pandemic [1]. In the breeding industry, goose parvovirus often causes huge economic losses due to large-scale epidemic in different regions [2]. Pregnant women are susceptible to human parvovirus B19, and it can be transmitted to the fetus after infection causing aplastic anemia[3–5]. Parvoviruses are important pathogens leading to animal diseases and have a significant role in gene therapy, oncolytic therapy, vaccine development, and as passive immune vectors [6].

Replication of progeny viruses in the nucleus is required for virus infection of eukaryotic species. Usually, viral genes pass through the cell membrane to reach the cytoplasmic region in a capsid-protected form, via intracytoplasmic RanGTP and other proteins are transported along microtubules (MTs) near the nuclear membrane and cross the nuclear membrane for progeny virus replication [7, 8]. The viral capsid enters the nucleus through the nuclear pore complex (NPC). The complex basket structure embedded in the inner and outer nuclear membranes mainly comprised the cytoplasmic ring, nucleoplasmic ring, nuclear basket, and other structures [9]. NPC can be regarded as a unique transmembrane transport protein complex that controls the movement of substances into and out of the nucleus with a bifunctional and a bidirectional hydrophilic nucleocytoplasmic exchange channel. The NPC is mainly composed of structurally nucleoporins. About half of the nucleoporins contain helical protein domains, while the other with typical “natively unfolded” structural features (high flexibility and lack of ordered tertiary structures) [10]. Some proteins cannot freely flow through it due to the restriction of its pore size, which has an inner diameter of roughly 39 nm in its spatial structure. In general, biological macromolecules can enter the nucleus via passive diffusion if their molecular weight is less than 40 kDa [11, 12]. In comparison macromolecules with molecular weight more than 40 kDa can only be actively transported through the NPC [13]. For example, herpes simplex virus type 1 (HSV-1) disintegrates on the cytoplasmic side, requiring direct or indirect interaction with NPC. The majority of the released viral genome is still attached to the nucleophilic virus protein, which is then actively introduced (such as the adenovirus genome), or the genome crosses the NPC (HSV-1) by repulsive force when the capsid is opened [14]. However, there is also a description of the passive diffusion of larger molecular weight molecules through the NPC. Timney et al. (2016) constructed a complex dynamic model to elucidate passive diffusion affected by factors such as the molecular mass and volume of the receiving substrate [15].

At present, the classical model is the mainstream nuclear transduction hypothesis. Recently, a novel paradigm proposed that importin-beta (impβ) alone mediates viral capsid passage through the nuclear envelope [16]. In 2022 Mattola et al. at the University of Jyväskylä, Finland discovered that parvovirus nucleation follows a new pattern [17]. This pattern involves the temporary dissolution of the nuclear envelope while Imp acts, allowing the genome to pass through. Below, we describe the transport of parvovirus and its capsid after enter into nucleus, and several modes of entry are highlighted.

## Overview of parvovirus

Parvoviruses (*Parvoviridae* family) are divided into three subfamilies: *Densovirinae*, *Hamaparvovirinae*, and *Parvovirinae*. The *Parvovirinae* subfamily is divided into 10 genera, including *Parvovirus*, *Erythrovirus*, and *Dependovirus*. The viruses of this family are round or hexagonal and non-enveloped, with 18-26 nm in diameter [18] and (5.5–6.2)×10^6^ in molecular weight, which composition is 75% of proteins and 25% of DNA. Some parvovirus VP1-specific regions contain an enzymatic catalytic center for phospholipase A2 (PLA2). VP2 and VP3 are proteins with antigenic properties and invasive cells. In addition, spermidine, arginine and butane diamine hydrochloride are also found in the virus particles. There are no lipids or glycosylated protein components [19].

Parvoviruses are highly inefficient compared to other viruses requiring only a few viral particles for infection [8]. In infection and disease development, the loss of this ability is compensated by a high degree of replication [20]. Because the ratio of particles to infectious units is very low, it is difficult to identify and follow the viral induction process, contributing to the spread of viruses. In addition, the small size of parvoviruses (approximately 20 nm in diameter) hinders the attachment of fluorescent probes [21], thus limiting the ability of single-virus tracking when detecting capsids.

## Cytoplasmic transport of parvovirus

### Microtubule-mediated transport mechanisms

MTs are important components of animal cytoskeletons, transporting various cell substances. Alpha and β tubulin heterodimers form the structure of MT in a rostrocaudal-manner to form protofilaments [22]. Thirteen protofilaments assemble around a hollow core to form a single microtubule filament, and heterodimers assemble to form two distinct ends, namely, α tubulin-linked microtubule minus ends and β tubulin-linked microtubule plus ends. Microtubule minus ends are usually fixed at their nucleation positions and the microtubule organization centers [23].

In recent years, with the discovery of various studies, the role of MTs in cells has become increasingly apparent. In the virus infection process in host cells, MTs provide: a dynamic skeleton for the interaction process between viruses and hosts and help viruses transport the proteins, nucleic acids, and other substances required for their replication in host cells. Adeno-associated virus (AAV) [24]is a member of the *Parvoviridae* family, which require helper viruses to complete the infection of cells. Pingjie Xiao and Samulski [25] discussed the effect of MTs on AAV-infected cells in their study. In the experiment, the involvement of MTs in the infection process of AAV was determined via live-cell imaging and flow cytometry. Meanwhile the subsequent validation of the infection process of AAV through quantitative 3D microscopy and PCR required the involvement of intact MT structures. The results showed that AAV2 was rapidly and unidirectionally transported in the perinuclear regions of the inner compartment through MTs after internalization, where the acidification of these membranous structures and viral escape took place. However, Hirosue et al. found no effect of AAV2 transduction on nocodazole-treated MT-ruptured Hela cells using FACS [26]. Both dynein-mediated and microtubule-independent pathways can lead to efficient rAAV2 transduction.

### Relying on ran protein-assisted transport

RAN is a nucleocytoplasmic shuttle protein involved in cell cycle regulation, nucleocytoplasmic transport, and cellular transformation and functions to support the transport between the cell interior and nuclei [27], which is a member of the Ras GTPase family [28]. Most studies are remained on impβ1-mediated nuclear transport of viral proteins, while further studies on whether Ran-dependent assistance is lacking. Yudin. D [29] proposed a new model in his experiments, through which it was found that in uninjured axons, a complex formed by RanGTP with CAS (Crm1-Associated Protein), Impα, and dynein was observed. RanBP1 and RanGAP promote the hydrolysis of RanGTP to RanGDP, which is expressed at low levels in uninjured axons, but their concentration in the axonal cytoplasm increases significantly after nerve injuries. Such an upregulation is achieved through the local translation of axonal Ranbp1 mRNA and RanGAP mechanism. Newly-synthesized RanBP1, together with RanGAP, promotes the dissociation of Ran from the importin-α-dynein complex in axons and hydrolyzes RanGTP to GDP, allowing impα to be bound on dynein via newly-translated impβ1, thus creating a retrograde damage signaling complex ready to bind to cargo. Unlike classical models with nuclear trafficking, this mechanism may require the local production of RanGTP in axonal cytoplasm, possibly through axonal RanGF. In vivo, the system is perturbed by introducing a dominant-negative Ran mutant or the addition of blocking antibodies against Ran or RanBP1, which in part reveals a novel function of Ran in the regulation of cytoplasmic transport.

The Ran-mediated transport mechanism was confirmed in an experiment of Li.M [30] in which a significantly negative (DN) RanGTP (Ran-Q69L) deficient in GTP hydrolysis was experimentally introduced, which was rendered deficient in determining whether the nuclear trafficking of UL54 required Ran. COS-7 cells were co-transfected with UL54-EYFP and Ran-Q69L-ECFP, whose subcellular localization patterns were monitored. As a result, the nuclear import of UL54 was significantly blocked due to the co-transfection of Ran-Q69L, indicating that the nuclear translocation of UL54 was Ran-dependent and required GTP hydrolysis. Therefore, it can be shown that the Ran protein family plays an important role in cytoplasmic transport [31].

## Several modes of nuclear transduction

### Classical nuclear transduction pathways

**Fig. 1:**
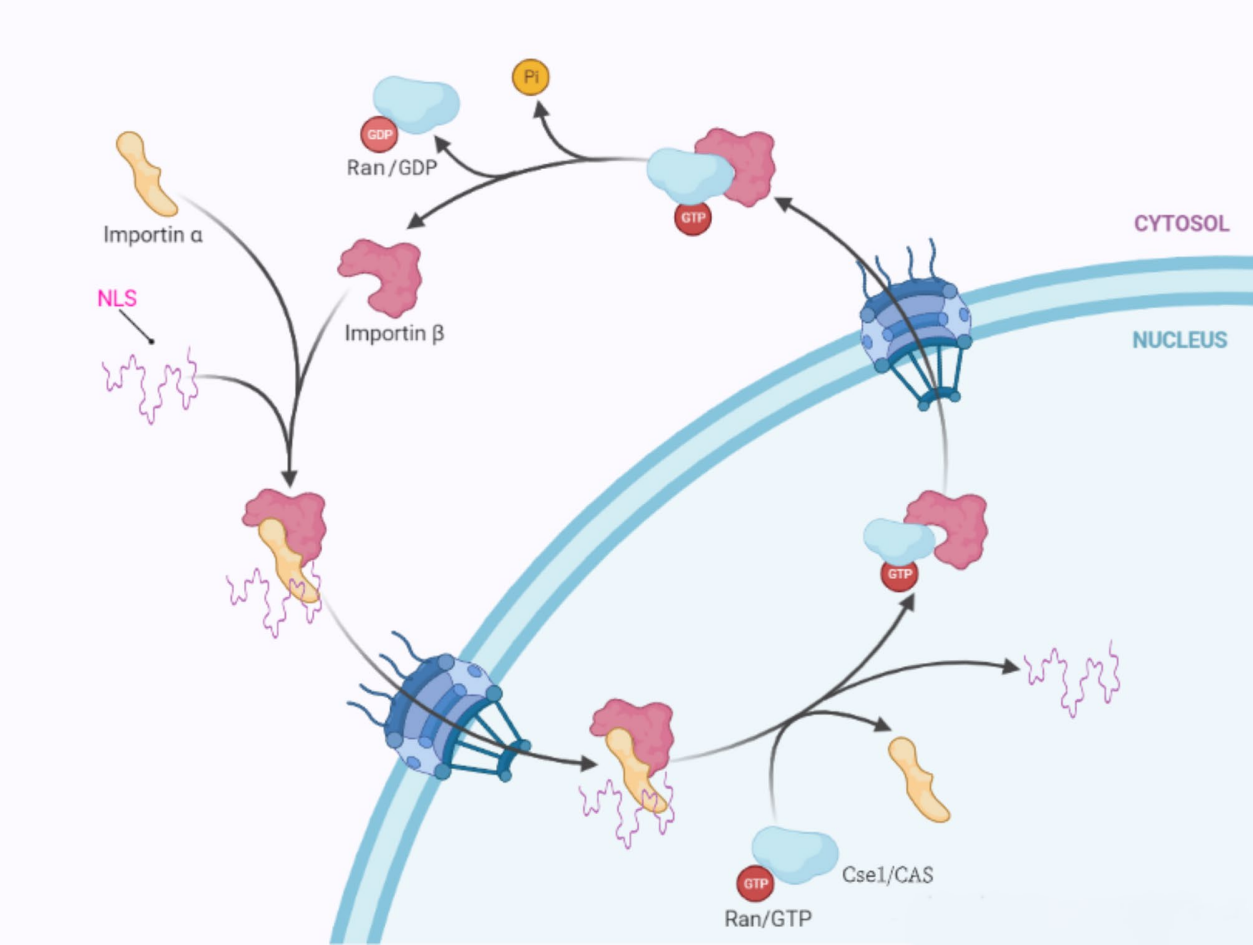
The classical nuclear transduction pathway co-mediated by impα and impβ. The linear fraction is the virus that has invaded the cells bound to the NLS. Forms trimeric complexes with impα and β through the nuclear pore complex. The detailed process is presented in the text

Classical nuclear transduction pathways mainly rely on impα acting with impβ, which is a famous adaptor protein that acts as a connector during protein-nuclear import [32]. Through impα, the nuclear import protein, lysine-rich NLS, can be recognized, which can be attached to the C-terminus of impβ through its N-terminal IBB (impβ binding) region; that is, substrate proteins with NLSs can be recognized via impα during nuclear import, which then connects with impβ to form a trimeric complex [33] docking on one side of NPC through the interaction of impβ and NPC proteins, followed by trafficking into the nuclei through NPC. Under the action of small molecule Ran-GTPase (GTPase Ran) in the nucleus, the “impα/impβ1 input protein-substrate” is depolymerized, the substrate is released into the nuclei, in which impβ1 is bound to RanGTPase, resulting in the release of α input proteins and the binding to the nuclear export protein Cse1/CAS, which returns to the cytoplasm through NPC with the participation of Ran-GTPase. Meanwhile Ran-GTP is hydrolyzed to Ran-GDP in cytoplasm so that impα is released in cytoplasm and participates in new transport processes as shown in Fig. 1.

### Two nucleation pathways mediated by importin β

Import proteins (importins), also known as karyopherins are receptor proteins of nuclear localization signals, which are responsible for nuclear import as importins and nuclear export as exportins. They are distributed in the cytosol of eukaryotic organisms and are relatively conserved, which have two subunits, α and β. They transport transcription factors, splicing factors, and other proteins in the cytoplasm to proteins in nuclei through the nuclear pore complex [34].

**Fig. 2:**
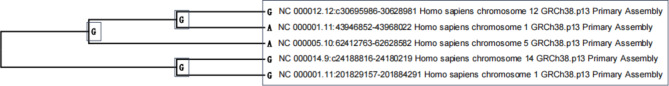
Evolutionary tree of several human impβ species. Homo sapiens differed less between chromosome 14 and chromosome 1, and more between chromosome 5 and chromosome 12

**Fig. 3:**
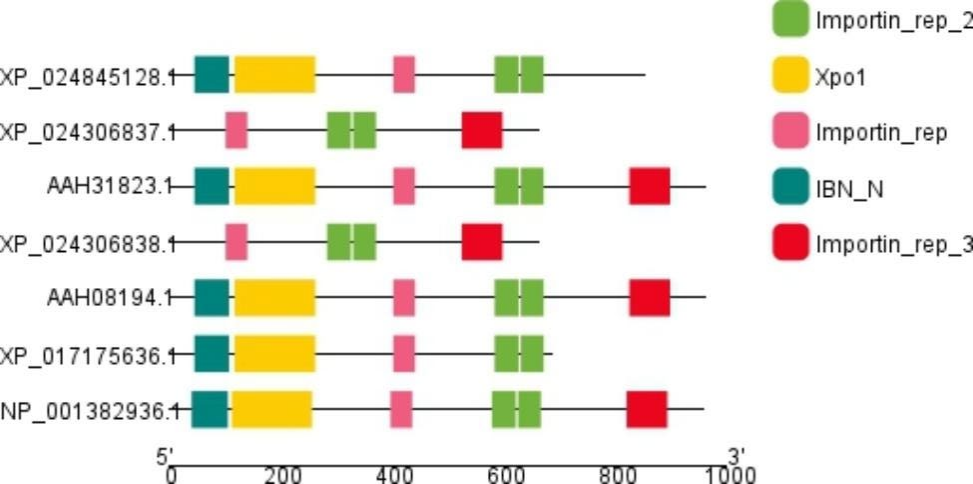
Schematic diagram of the structure of importin β protein of different species

To date, 14 impβ family members have been found in yeasts, 22 in humans, and a large number in other mammals, as shown in Fig. 2. The profile suggests that these complex responses can be stably completed in animals with more complex higher-order vital responses and more imp β families in their bodies. These impβ proteins are relatively functionally-conserved [35], as is shown in Fig. 3. Their protein sequence similarity is low, with a molecular weight of 94-145kda [36], all of which have a conserved N-terminal Ran-binding domain (IBN-N Domain) [37] and multiple HEAT repeat domains at the C-terminus [38]. The heterogeneity of Ran-binding domains is prominent, with a sequence similarity of only approximately 10% to Ran-binding domains throughout the superfamily, and no invariant residues are found in the core motifs bound to Ran [39]. Through this particular structure, impβ family members can bind to substrates or adaptor proteins using the C-terminus, to RanGTP through the N-terminus, and nucleoporins in the middle, thereby bringing substrates into or out of nuclei. Through a crystal structure analysis of impβ, it has been found that its HEAT repeat supercoiled domain can stretch or contract, changing various conformations to match structurally-different substrates.

#### Impβ directly mediates the nuclear entry pathway

**Fig. 4:**
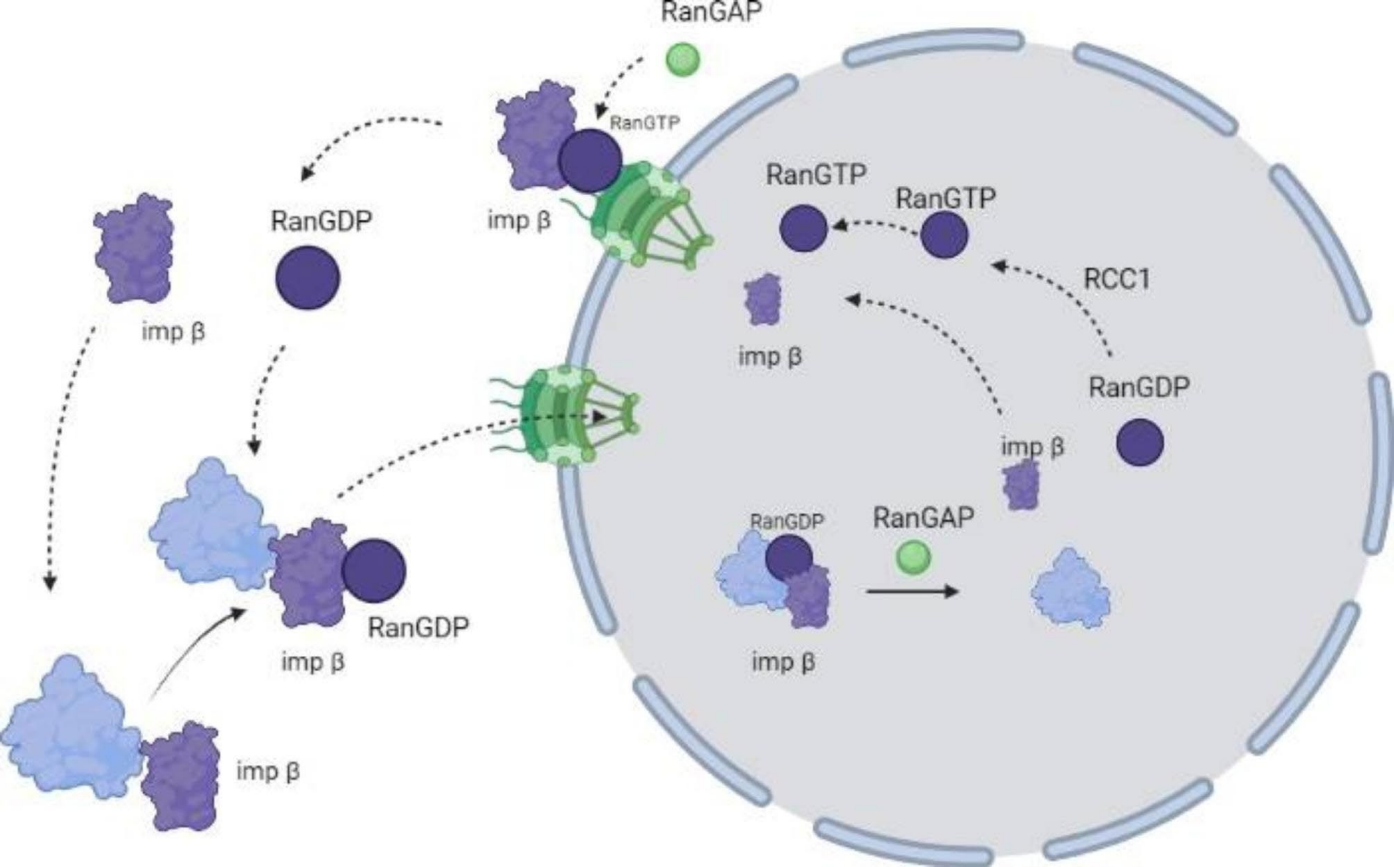
A parvovirus nuclear entry pathway mediated by the combined action of Impβ and RanGTP. The purple irregular graph is impβ; the blue irregular shape is the viral capsid

Recently, it has been found that impβ can directly guide the cytoplasmic transport process of viral capsids where impα is not involved [40]. As is shown in Fig. 4, the nuclear localization sequence and the target protein NLS are also recognized via impβ1 in the cytoplasm, which is bound to form an impβ1-target protein binary complex and a target protein-importin β1-RanGDP ternary complex with it through the mediation of RanGTP proteins and traffics to nuclear membranes [41]. Subsequently, RanGDP in the complex binds to nucleoporin nuclear transport factor 2 on NPC, resulting in a conformational change in NPC that dislocates nucleoporins initially located on the outer side of nuclei to their inner side when the ternary complex bound to nucleoporins enters nuclei. Since RanGDP in nuclei is replaced by RanGTP with a higher concentration to form a new target protein-importin β1– RanGTP ternary complex, RanGTP bound to the β subunit of importin β1 is hydrolyzed to RanGDP via RanGAP, and the new ternary complex is dissociated into a target protein, importinβ1–RanGDP, through the released energy. The target protein remains in nuclei, and RanGTP replaces RanGDP in importinβ1-RanGDP to form an importinβ1-RanGTP complex in response to the chromosome condensation regulator RCC1 while penetrating NPC into cytoplasm, where importinβ1-RanGTP is hydrolyzed to importinβ1 and RanGDP through RanGAP. At this time, newly-synthesized or dissociated importinβ1 and RanGDP in cytoplasm wait for a new round of nuclear transport of target proteins.

#### Cytoplasmic parvovirus capsid recruitment of impβ for nuclear delivery

**Fig. 5:**
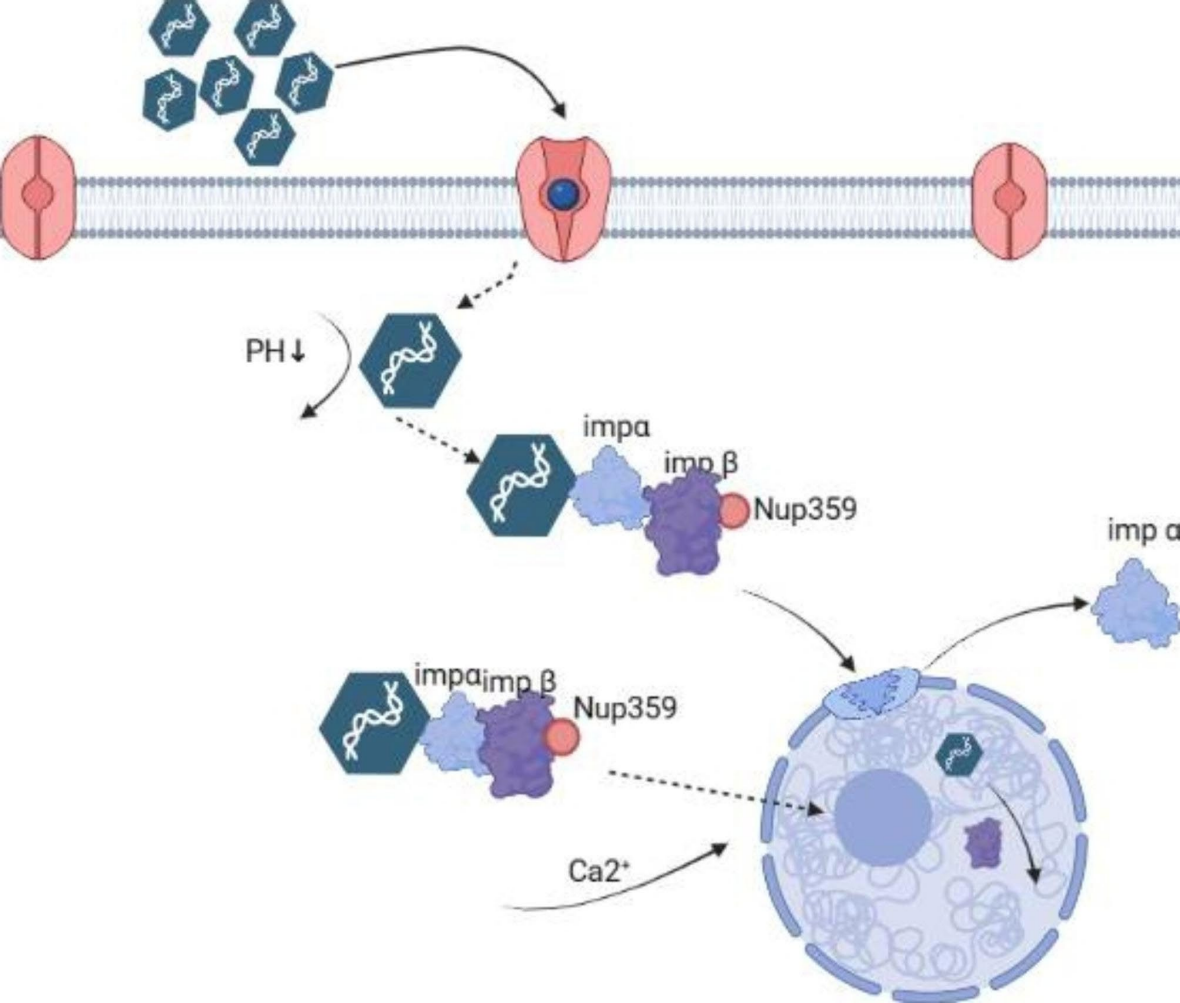
Schematic diagram of parvovirus capsid entry and nuclear entry. The capsid is shown as a polygon, with dotted lines indicating the entry pathway of nuclear film degradation. Further details are presented in this paper. Purple for impβ; blue is impα

Mäntylä et al., 2018 and 2020 [14] neutralized impβ with antibodies by microinjection through canine parvovirus (CPV) entry nucleus experiment The statistical result of CPV-infected cells showed that the number of infected cells was few, which verified that impβ plays an important role in the process of the virus entering and crossing the nuclear membrane. Subsequently, co-immunoprecipitation and in situ proximity ligation assay (PLA) experiments were carried out. The results of PLA experiments revealed that the number and density of signals close to the nuclear envelope(NE) were the highest in the nucleus. A small part of the signals was also located deeper, 0.75 ~ 6.0 m away from the NE. Quantitative analysis showed that the capsid enters the nucleus before interacting with the cytoplasmic impβ. Subsequently the capsid enters the nucleus, unlike classical nucleophiles, where the capsid still interacts with Impβ. Based on the phenomenon of nuclear permeability of viral capsids as it enters the nucleus observed in other viruses. Figure 5 illustrates a theory that the viral capsid’s structure change due to internal acidification after entering the cell, resulting in exposure of the VP1 N-terminal domain, which contains phospholipase A2 and NLS on the capsid surface [42, 43]. The phospholipase activity disassembles the endosomal membrane, while the NLS interacts with impα and β after the endosome opens. Alternatively after capsid release from endosomes during capsid transport using MTs [25, 44–46]. Impβ binds to the NPC and passes through the pore. The process is also observed during CPV nucleation. The portion of the capsid that impβ does not mask interacts with Nup358, Nup62, and Nup153. In anticipation of the interaction of impβ with Nup153 to terminate translocation in the nuclear basket, RanGTP dissociates and imports into the complex [47, 48], leading to the recycling of Impβ into the cytoplasm. At the same time, NPC-bound capsids disintegrate the NPC/NE, possibly because impβ regulates cell mitosis [49] and penetrates the nuclear membrane, triggering Ca_2_^+^ release, a known initiator of NE degradation in mitosis. Subsequently, the cytoplasmic capsid-imp complex can then passively enter the nucleus thanks to the pore in the NE. This is a novel hypothesis about the nuclear pathway of the parvovirus genome (CPV), classical nuclear import through the nuclear pore complex, accompanied by instantaneous rupture of the nuclear envelope, which also allows passive entry of the protein capsid complex into the nucleus. The low transduction/infection efficiency could not be fully explained by the restriction of virus-receptor (transferrin receptor, a transferrin receptor on the cell membrane surface of canine parvovirus) [50] interaction or endocytic entry, indicating a bottleneck occurs after viral entry into the cytoplasm. Therefore, this hypothesis can be a relatively accurate and complete elaboration of the parvovirus nucleation process.

#### Disease treatment application of impβ with acute viral infection identification application

Inhibition of viral entry into cells is a major area of research in virology, and inhibition of viral infection is achieved by preventing the binding of the viral capsid to the ligand. In the case of the Impβ protein, this can also be achieved by inhibiting its activity or reducing its levels in the cytoplasm, such as by neutralising the Impβ antibody. The nuclear transport receptor impβ/karyopherin-β1 is overexpressed in cancers with unstable genomes, according to Verrico [49]. Inhibitors are being developed since it is considered a promising cancer target. Impβ regulates mitosis, and it functions in nucleocytoplasmic transport [49]. Therefore, using impβ inhibitors to treat cancer is also considered a more feasible strategy. However, over expression of impβ might be of interest for treatment with an oncolytic virus (H1) or vector (AAV), so inhibiting impβ would not be a good approach to treat this tumor. Nuovo [51] pointed out that impβ and extin-5 are indicators of acute viral infection and immunohistochemical markers in experiments. Virus-infected cells were detected by in situ-based methods (reovirus, rabies virus) or cytological changes (molluscum contagiosum virus, herpes simplex virus). Impβ and Exportin-5, two proteins involved in nuclear transduction, were detected in infected cells of each virus, but not in control tissues. Various other proteins, including Caspase-3 and Bcl-2 family members (Bcl2, BclX, etc.) showed vast differences in expression between different viral infections. The specificity of impβ and extin-5 signals vary greatly, with different commercially available peroxidase conjugates. So immunohistochemical detection of Impβ and extin-5 may be valuable markers of acute viral infection, indicating that the imposition of nuclear transduction may be an crucial concomitant factor for viral proliferation.

## Summary and prospect

With some experimental data, a more credible process can be obtained that is different from the whole previous process of parvovirus entering the nucleus. However, some problems still cannot be explained at this time. Whether the NPC-bound or the free nuclear capsids initiate infection cannot be explained. According to the characteristics of phylogenetically distant viruses such as HTLV-1 and papillomaviruses, which require NE permeabilization to enter the nucleus during mitosis, such as adenovirus and herpesviruses bind to NPCs, triggering genome release and subsequent genome pass through nuclear pores [14]. It is optimistic about emerging experimental techniques, such as NGS (Next-generation sequencing, NGS) and fluorescence imaging based on research over the past few decades. These two techniques allow detailed analysis of infection-induced changes in host chromatin organization and high-resolution imaging of parvovirus infection. Combined with the current popular spatial transcriptomics technology, we can analyze the spatial heterogeneity from the level of gene expression.

NGS is a modern sequencing method with massively parallel sequencing to map the sequence of millions of small DNA fragments. The obtained sequencing data were then combined using bioinformatics, and compared with a reference genome. The information about expressed genes [52], genome accessibility [53], binding regions of different DNA interacting proteins [54, 55], or chromatin-chromatin interactions and organization were obtained. For example, Transposase Accessible Chromatin Sequencing (ATAC-SEQ) is based on a highly active Tn5 transposase mutant [53]. Salla Mattola [56] utilized highly active Tn5 to attach short and specific DNA oligomers to accessible regions, thereby marking accessible chromatin. These genome regions are then isolated and sequenced, generating high-resolution maps of accessible genome regions. Therefore, ATAC-seq has great potential for studying how parvovirus infection alters the packaging or release of host cell chromatin organization or viral genomes.

In the study of parvovirus nucleation, many serious parvovirus nucleation mechanisms have not been significantly broken through, such as human parvovirus B19, porcine parvovirus in livestock breeding, and feline parvovirus causing feline panleukopenia Currently, most studies focus on the involvement of impβ protein in nuclear transduction with the traditional classical pathway, little new advances have been made on other proteins. Viral nucleation is a complex process in which many key proteins are involved. Among them, impα is the connector in classical nuclear transduction mode, which can identify approved micro signals and cooperate with impβ to complete classical nuclear transport. However, it was found that impα could also independently mediate the nucleation of viral genetic information, such as (Vpr) human immunodeficiency virus type I. There are few studies on the involvement of the Ran protein, and it can be found from the relevant literature that the Ran family plays an important role in regulating nuclear membrane and cytoplasmic transport. The Ran family is closely related to the spread of cancer cells. Presently, the relevant applications of impβ have been fruitful, for example, using drugs such as impβ inhibitors to prevent the virus from entering the nucleus to treat related diseases and using impβ as the main component of the detection kit. Both perspectives are relatively novel. However, there is still a phenomenon of substandard sensitivity. Future research hotspots may be developing new targeted drugs, and positioning of hallmark proteins. There is also an urgent need to apply related detection technologies in the poultry farming industry.

## Data Availability

All data generated or analysed during this study are included in this published article.
